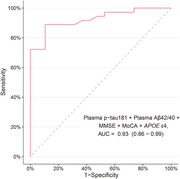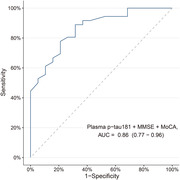# Development, validation, and diagnostic evaluation of an integrated model for brain Aβ pathology in early stage of Alzheimer’s Disease: a multicenter study in China

**DOI:** 10.1002/alz.095469

**Published:** 2025-01-09

**Authors:** Zhibo Wang, Qihao Guo, Bote Zhao, Qin Chen, Xia Li, Lu Shen, Jianping Jia

**Affiliations:** ^1^ Xuanwu Hospital, Capital Medical University, Beijing, Beijing China; ^2^ Shanghai Sixth People’s Hospital Affiliated to Shanghai Jiao Tong University School of Medicine, Shanghai China; ^3^ Xuanwu Hospital, Capital Medical University, Beijing China; ^4^ West China Hospital of Sichuan University, Chengdu China; ^5^ Shanghai Jiao Tong University School of Medicine, Shanghai, Shanghai China; ^6^ National Clinical Research Center for Geriatric Disorders, Xiangya Hospital, Central South University, Changsha China; ^7^ Innovation Center for Neurological Disorders, Xuanwu Hospital, Capital Medical University, Beijing, China;, Beijing China

## Abstract

**Background:**

There is an urgent need for accurately predicting amyloid‐beta (Aβ) pathology in Alzheimer’s disease (AD) using non‐invasive and readily available methods, particularly in the early stages of the disease. While blood‐based biomarkers have shown promising consistency with brain Aβ pathology, there remains a scarcity of data predicting Aβ pathology using these biomarkers across different clinical settings in the Chinese population.

**Method:**

We retrospectively selected 278 participants from five hospitals across China, comprising 127 patients with mild cognitive impairment (MCI) and 151 with dementia. All participants underwent amyloid PET scanning, cerebrospinal fluid (CSF) tests, and plasma analysis for Aβ42/40 ratios, phosphorylated tau181 (p‐tau181), neurofilament light chain (NfL), and APOE ε4 genotype, alongside cognitive assessments using the MMSE and MoCA. Patients were categorized as Aβ positivity based on amyloid PET or CSF Aβ42/40 ratio results, with 171 classified as Aβ positivity. Predictive models were constructed using the Random Forest algorithm for feature selection and model building. Participants were divided into a training set (n = 223) and a testing set (n = 55) for model development and validation, respectively.

**Result:**

Two models were developed to differentiate Aβ positive from Aβ negative patients. The high‐accuracy model, incorporating plasma p‐tau181, plasma Aβ42/40 ratios, APOE ε4 status, MMSE, and MoCA, demonstrated excellent predictive capability with an area under the receiver‐operating characteristic curve (AUC) of 0.93 (95% CI 0.86–0.99) (Figure 1). A more parsimonious model including only plasma p‐tau181, MMSE, and MoCA also showed high accuracy (AUC: 0.86 [95% CI 0.77–0.96]) (Figure 2) in identifying patients with Aβ positivity. Both models were validated across participating centers and outperformed individual plasma biomarkers. Additionally, these models exhibited similar performance among participants with or without comorbidities or populations that included cognitively normal individuals.

**Conclusion:**

The developed models, utilizing plasma biomarkers and clinically accessible parameters, provide a non‐invasive, cost‐effective approach for predicting brain amyloid positivity in the early stages of AD. This approach has potential to enhance early screening, clinical diagnosis, and the initiation of disease‐modifying therapies within the Chinese context.